# Simeprevir with pegylated interferon alfa 2a plus ribavirin for treatment of hepatitis C virus genotype 1 in patients with HIV: a meta-analysis and historical comparison

**DOI:** 10.1186/s12879-015-1311-3

**Published:** 2016-01-11

**Authors:** Frank Andersohn, Anne-Kathrin Claes, Werner Kulp, Jörg Mahlich, Jürgen Kurt Rockstroh

**Affiliations:** 1 Charité – University Medicine Berlin; Institute for Social Medicine, Epidemiology and Health Economics, 10098 Berlin, Germany; 2 Frank Andersohn Consulting & Research Services, Mandelstr. 16, 10409 Berlin, Germany; 3 Xcenda GmbH, Lange Laube 31, 30159 Hannover, Germany; 4 Janssen K.K., Tokyo, Japan; 5 University of Düsseldorf; Düsseldorf Institute of Competition Economics (DICE), Universitätsstraße 1, 40225 Düsseldorf, Germany; 6 Department of Medicine I, University Hospital Bonn, Sigmund-Freud-Str. 25, 53105 Bonn, Germany

**Keywords:** Chronic hepatitis, Hepatitis C virus, Human immunodeficiency virus, Simeprevir, Pegylated interferon alfa, Ribavirin, Efficacy, Safety, Historical comparison

## Abstract

**Background:**

About one third of patients infected with human immunodeficiency virus (HIV) also have chronic hepatitis due to hepatitis C virus (HCV). HCV therapy with simeprevir, pegylated interferon alfa (PegIFNα) and ribavirin (RBV) have been shown to be superior to PegIFNα + RBV alone in non-HIV patients, but no randomized trials in patients with HCV genotype 1 (HCV-1) / HIV coinfection are available.

**Methods:**

This was a historical comparison of study C212 (simeprevir + PegIFNα-2a + RBV in patients with HCV-1/HIV coinfection) with studies in which HCV-1/HIV coinfected patients were treated with PegIFNα-2a + RBV alone. A systematic literature search was performed to identify eligible studies. Efficacy and safety results of PegIFNα-2a + RBV studies were combined in random- and fixed-effects inverse-variance weighted meta-analyses of proportions using the Freeman-Tukey double arcsin transformation method, and compared with the results of study C212.

**Results:**

The literature search revealed a total of 2392 records, with 206 articles selected for full-text review. Finally, 11 relevant articles reporting on 12 relevant study groups were included. Results on sustained virologic response 24 weeks after end of treatment (SVR24) were available from all 12 study groups. Pooled SVR24 for PegIFNα-2a + RBV from the random-effects meta-analysis was 28.2 % (95 % CI 23.8 % to 32.9 %). The comparison between study C212 (SVR24 = 72.6 %; 95 % CI 63.1 % to 80.9 %) revealed substantial superiority of simeprevir + PegIFNα-2a + RBV compared to PegIFNα-2a + RBV alone, with an absolute risk difference of 45 % (95 % CI 34 to 55). This finding was robust in a sensitivity analysis that only included historical studies with a planned treatment duration of at least 48 weeks and the same RBV dose as in study C212. No increases in the frequency of important adverse event categories including anemia were identified, but these analyses were limited by the low number of studies.

**Conclusion:**

This historical comparison provides first systematic evidence for the superiority of simeprevir + PegIFNα-2a + RBV compared to PegIFNα-2a + RBV in patients with HCV-1 / HIV coinfection. Given the limitations of the historical comparison for safety endpoints, additional data on the comparative safety of simeprevir in patients with HCV-1 / HIV coinfection would be desirable.

**Trial registration:**

Identifier for study TMC435-TiDP16-C212 (ClinicalTrials.gov): NCT01479868.

**Electronic supplementary material:**

The online version of this article (doi:10.1186/s12879-015-1311-3) contains supplementary material, which is available to authorized users.

## Background

Chronic hepatitis due to hepatitis C virus (HCV) is an important comorbidity in people infected with the human immunodeficiency virus (HIV). It has been estimated in US [1] and European [2] studies that approximately one third of patients with HIV are coinfected with HCV. The course of chronic hepatitis due to HCV has been reported to be more severe in people with HIV, leading to an increased risk of decompensated liver disease, histological cirrhosis [3, 4] and death [4]. The increased duration of survival in people with HIV due to the efficacy of highly active antiretroviral therapy (HAART) has additionally augmented the importance of successful HCV treatment.

A combination therapy of pegylated interferon alfa (PegIFNα) plus ribavirin (RBV) has been the cornerstone of HCV treatment for several years. During the last years, several new direct acting antivirals have been developed or are currently in phase II or phase III studies. Three NS3/4A serine protease inhibitors (boceprevir, telaprevir and simeprevir) have been licensed for the treatment of HCV genotype 1 (HCV-1) infection in combination with PegIFNα + RBV. In individuals without HIV infection, adding these drugs to PegIFNα + RBV has substantially increased HCV eradication, measured by sustained virologic response (SVR = undetectable HCV RNA 12 (SVR12) or 24 (SVR24) weeks after completion of treatment) [5]. One randomized controlled trial (RCT) evaluating the efficacy and safety of these drugs in patients with HCV-1/HIV coinfection is available for boceprevir [6] and telaprevir [7], respectively. However, for simeprevir which has been reported to have advantages over boceprevir and telaprevir in terms of pill-burden and adverse event (AE) profile, only a single-arm trial (study identifier TMC435-TiDP16-C212 = „study C212“) is available [8]. A systematic comparison between simeprevir + PegIFNα-2a + RBV vs. PegIFNα-2a + RBV alone in patients with HCV-1/HIV coinfection is, however, lacking. Aim of this study was to compare the main results from study C212 (simeprevir + PegIFNα-2a + RBV) with data from earlier (historical) studies that evaluated the efficacy and safety of PegIFNα-2a + RBV in patients with HCV-1/HIV coinfection. Focus of this comparison was SVR, in addition data on important AE categories (e.g. total AEs; total SAEs; total AEs leading to discontinuation) were also extracted for the comparison.

## Methods

### Study design

This is a historical comparison (also called non-adjusted indirect comparison) which compares study C212 (simeprevir + PegIFNα-2a + RBV in patients with chronic HCV-1/HIV coinfection) with studies and/or study groups in which HCV-1/HIV coinfected patients were treated with PegIFNα-2a + RBV only. The conduct of the historical comparison consisted of the following steps: 1) a systematic literature search and selection of relevant studies; 2) a meta-analysis of proportions; 3) a re-transformation of the meta-analysis results (i.e. proportions and 95 % confidence intervals (CI)) into corresponding numerator / denominator pairs; and 4) a historical comparison of the results from study C212 with historical results from the meta-analysis. The systematic review and meta-analysis was performed and reported in accordance with the Preferred Reporting Items for Systematic Reviews and Meta-Analyses (PRISMA)-Statement [9].

### Eligibility criteria

To increase the validity of the historical comparison inclusion and exclusion criteria were aligned to the study design of C212. Study C212 (ClinicalTrials.gov identifier NCT01479868) is an open-label, single arm clinical study to evaluate the safety, tolerability and efficacy of simeprevir along with PegIFNα-2a and RBV triple therapy in adult chronic HCV genotype-1 infected patients who were coinfected with the human immunodeficiency virus-type 1 (HIV-1). The study included HCV treatment-naïve patients who never received medication for the treatment of HCV, as well as relapsers and non-responders to prior HCV therapy . All patients received Simeprevir 150 mg q.d. for 12 weeks, in combination with PegIFNα-2a (180 μg per week) and RBV (1000 mg b.i.d. for patients with a body weight <75 kg; and 1200 mg b.i.d. for patients with a body weight ≥75 kg). The duration of therapy with PegIFNα-2a and RBV was 24 or 48 weeks, depending on virologic response: A shortened duration of 24 weeks was used for treatment-naïve patients or relapsers who had a HCV-RNA <25 IU/mL at week 4 (detectable or non-detectable) and at week 12 (non-detectable); all other patients received 48 weeks of treatment.

To be included in the meta-analysis studies had to fulfill all of the following inclusion criteria: 1) Adult patients with chronic HCV genotype-1 and HIV coinfection. Studies with patients who had chronic hepatitis C due to other HCV genotypes were only included, if the publication included HCV-1 stratified results for at least one of the endpoints of interest. 2) Treatment with PegIFNα-2a and RBV for at least 12 weeks (for safety endpoint) or 24 weeks (for SVR). Studies involving PegIFNα-2b were excluded due to potential pharmacodynamics differences between PegIFNα-2a and PegIFNα-2b. 3) Results are presented for at least one of the pre-defined endpoints of interest: Proportion of patients with SVR 12 or 24 weeks after end of therapy; proportion of patients with at least one AE; proportion of patients with at least one serious AE; proportion of patients who interrupted treatment due to an AE; proportion of patients with AEs of special interest (i.e. anemia; psychiatric disorders; infections; increase of blood bilirubin levels; adverse cutaneous events). 4) Study was a RCT or another (e.g. single-arm) clinical trial, or an observational cohort study. 5) Study results were available from a full publication in a scientific journal or a study report (i.e. meeting abstracts and/or scientific poster presentations were not included). Studies were excluded, if the population was highly selected (e.g. selected by previous response/non-response to PegIFNα and/or RBV; selected by comorbidity such as hemophilia; selected by concomitant treatment such as a specific HIV therapy) or if the publication presented a case series or a study with a very low number of participants (i.e. N < 10 patients in the relevant analyses). For studies with more than one study arm (e.g. RCTs), the inclusion of two or more study groups were possible as long as they fulfilled the eligibility criteria.

### Systematic literature search and data extraction

The systematic search for relevant publications were performed at July 24, 2014, using the electronic databases Medline, EMBASE (Excerpta Medica Database) and the Cochrane Central Register of Controlled Trials. The search included terms for HCV and HIV, in combination with intervention terms for PegIFNα-2a and RBV. The search was limited “human” and to English or German language. The full electronic search strategy for Medline is included in an online appendix as an example (Additional file 1 - electronic search strategy for Medline). Selection of relevant studies or study groups were performed independently by two authors; potential discrepancies were resolved by discussion. For all included studies, pre-defined study design characteristics; characteristics of the study populations; and results of the studies (for relevant endpoints) were extracted.

### Statistical analysis

As all endpoints of interest were proportions (i.e. number of patients with a certain event divided by the total number of patients), an inverse-variance weighted meta-analysis of proportions using the Freeman-Tukey double arcsin transformation method [10, 11] was performed for each endpoint of interest. Both fixed- and random-effects meta-analyses were performed and heterogeneity was evaluated based on Cochran’s Q; I^2^; and the test for heterogeneity. To allow a direct comparison with study C212, the meta-analytical proportions including their 95 % CI were re-transformed into the best-fitting corresponding numerator/denominator pair using iteration. The iteration identified the numerator/denominator pair for which the width of the 95 % binomial (Wilson score) CI is most similar to the width of the meta-analytical 95 % CI. For example, for a meta-analytical proportion of 0.277 with a 95 % CI of 0.253 to 0.302, the best-fit numerator/denominator pair is 364/1313 (as it corresponds to a proportion of 0.277 with a 95 % Wilson CI of 0.253 to 0.302). Using these numerator/denominator pairs, relative risks as well as absolute risk differences of simeprevir + PegIFNα-2a + RBV (from study C212) vs. PegIFNα-2a + RBV (from the meta-analyses) were calculated for all endpoints of interest. If for an endpoint results from not more than one historical study were available, no meta-analysis was performed for that endpoint and the numerator/denominator pair from the single historical study was used for the comparison. To evaluate the potential impact of the duration of treatment and the daily dose of RBV on SVR24, a pre-planned sensitivity analysis was performed. This analysis included only studies with a treatment duration of 48 weeks for PegIFNα-2a + RBV and that used RBV in the same weight-based daily dose as in study C212. To allow a full evaluation of the available evidence a historical comparison of study C212 and each of the identified studies / study arms was performed in addition to the comparison with the results from meta analyses. All analyses were performed with SAS 9.3 (SAS Institute, Cary NC).

## Results

### Literature search

**Fig. 1:**
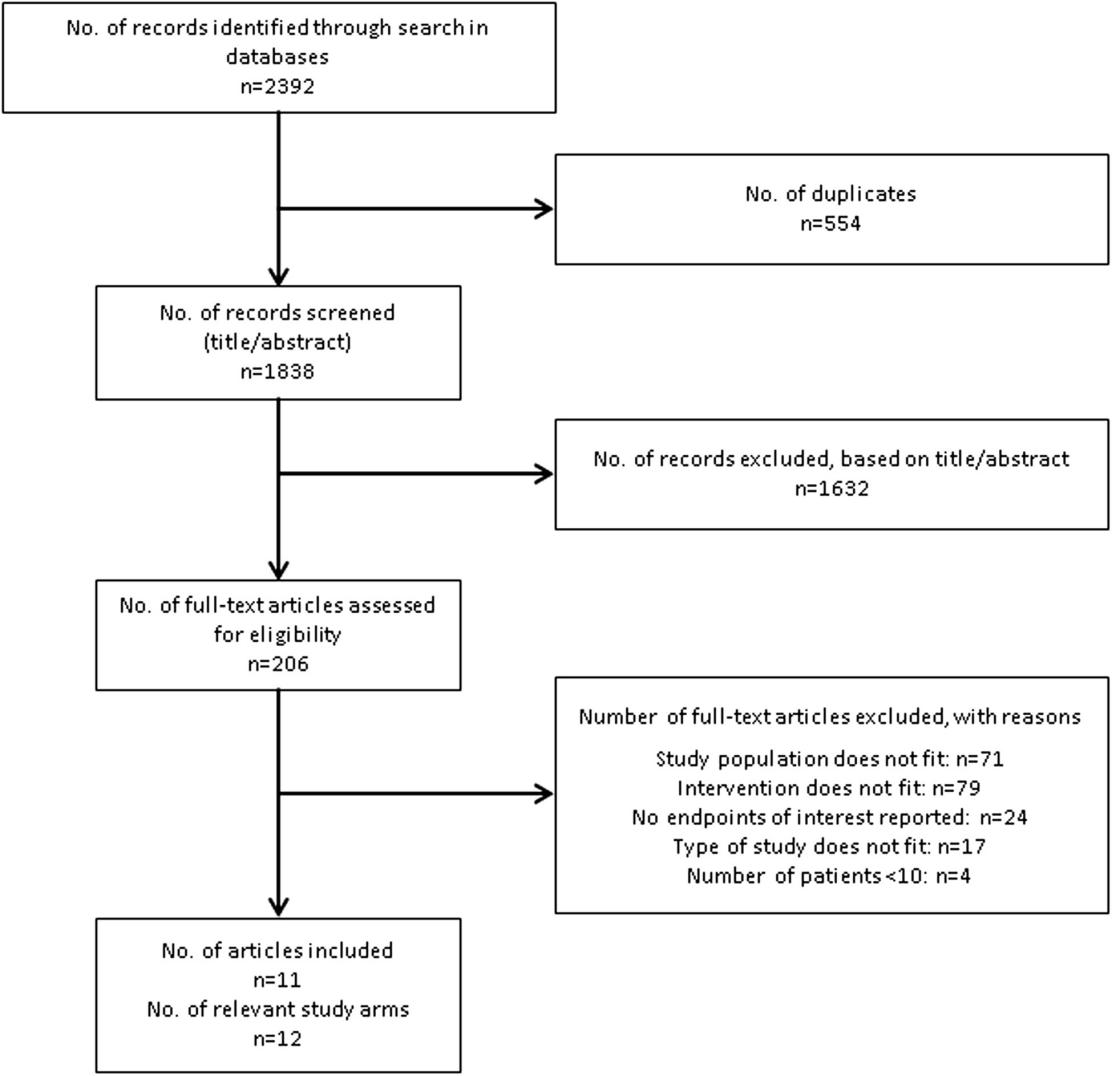
Flow chart of literature selection

The literature search in Medline, EMBASE, and the Cochrane Central Register of Controlled Trials revealed a total of 2392 records. After exclusion of 554 duplicates, a total of 1838 records remained for review. *N* = 1632 records were excluded based on title and/or abstract, leaving 206 articles for full-text review. From these publications, *N* = 195 articles were excluded while 11 relevant articles [12–22] were included (see Fig. 1 for details on the literature selection process). One of the 11 studies reported on two relevant study groups, while 10 had one relevant study arm, resulting in a total of 12 relevant study groups.

### Characteristics of included studies

The comparability between the included studies and study C212 with respect to important characteristics of study design and patient population was acceptable (see Table 1 for details). Differences were noted for the duration of treatment with PegIFNα-2a + RBV; the daily dose of RBV; and the inclusion/exclusion of relapsers and/or non-responders to previous PegIFNα-based HCV therapy. For the following endpoints of interest, results from at least one of the identified historical studies were available: Proportion of patients with SVR after 24 weeks (*N* = 12 study groups); proportion of patients with at least one AE (*N* = 2 study groups); proportion of patients with at least one serious AE (*N* = 2 study groups); proportion of patients who interrupted treatment due to an AE (*N* = 1 study group); proportion of patients with anemia (*N* = 3 study groups); and proportion of patients with psychiatric disorders (*N* = 1 study group). No historical comparisons were possible for SVR after 12 weeks and for the AE categories infections; increase of blood bilirubin levels; and adverse cutaneous events. All included historical studies reported on SVR24, while only a minor proportion reported on AEs. This was due to the fact that only results for patients with HCV-1 infection were of relevance for the historical comparison. Publications on studies that also included patients infected with other HCV genotypes, however, reported HCV-1 stratified results only for SVR but not for AEs.

**Table 1:** Characteristics of included studies

Study	Study period	Study region	Multi-center study?	Study design	Relapser/Non-responder included?	Patients on HIV therapy	Planned duration of treatment	Ribavirin dose per day	Number of patients^a^	Mean/median age (years)	Males (%)	Patients with liver cirrhosis (%)
Study C212	2011–2013	Multi-national	Yes	Single-arm	yes	85 %	Treatment-naïve patients + relapsers with EVR at week 4/12: 24 weeks	1000 mg / 1200 mg (w.a.)	*N* = 106	47 years	85 %	13 %
							All other patients: 48 weeks					
Chung 2004 [12]	2000–2002	US	Yes	RCT (not blinded)	no	82 %	Patients without response in week 24: 24 weeks	600 mg (week 1–4); 800 mg (week 5–8); 1000 mg (week >8)	*N* = 51	45 years	82 %	10 %
							Patients with response in week 24: 48 weeks					
Dahari 2010 [13]	n.r.	Brazil	No	Single-arm	n.r.	88 %	48 weeks	11 mg/kg body weight	*N* = 13	41 years	88 %	15 %
Fuster 2006 [14]	2001–2002	Spain	Yes	RCT (not blinded)	no	75 %	Patients with EVR in week 12: 48 weeks	800 mg	*N* = 51	39 years	75 %	39 %^b^
							Patients without EVR in week 12: 48 weeks or 72 weeks (randomized)					
Mandorfer 2014 [22]	n.r.	Austria	Yes	Single-arm	no	73 %	Patients with RVR in week 4: 48 weeks	1000 mg to 1200 mg for 12 weeks; 800 mg thereafter	*N* = 28	37 years	73 %	42 %
							Patients without RVR in week 4: 72 weeks					
Murphy 2011 [15]	2004–2007	US	No	RCT (not blinded)	n.r.	90 %	48 weeks	1000 mg / 1200 mg (w.a.)	*N* = 10	48 years	90 %	n.r.
Nunez 2007 [16]	2003–2006	Spain	Yes	Non-randomized study	no	78 %	Patients without EVR in week 12: 12 weeks	1000 mg / 1200 mg (w.a.)	*N* = 191	39 years	77 %	11 %
							Patients with detectable HCV in week 24: 24 weeks					
							All other patients: 48 weeks (since August 2004: 72 weeks)					
Rivero-Juarez 2014 [17]	n.r.	Spain	Yes	Non-randomized study	no	92 %	Treatment according to 2009 AASLD guidelines (48 weeks; patients with delayed virologic response: 72 weeks)	1000 mg / 1200 mg (w.a.)	*N* = 192	42 years	82 %	51 %^c^
Rodriguez-Torres 2012 [21]	2006–2009	US, Spain, Portugal	Yes	RCT (blinded)	no	89 %	48 weeks^d^	Treatment group 1: 800 mg	*N* = 135 (group 1)	45 years	80 %	12 %
								Treatment group 2: 1000 mg / 1200 mg (w.a.)	*N* = 275 (group 2)			
Torres-Cornejo 2014 [18]	2004–2011	Spain	No	Non-randomized study	no	85 %	48 weeks	1000 mg / 1200 mg (w.a.)	*N* = 135	41 years	85 %	39 %
Torriani 2004 [19]	2000–2003	Multi-national	Yes	RCT (blinded)	no	84 %	48 weeks	800 mg	*N* = 176	40 years	80 %	15 %
Tural 2008 [20]	2003–2005	Spain	Yes	Non-randomized study	n.r.	n.r.	48 weeks	1000 mg / 1200 mg (w.a.)	*N* = 55	40 years	67 %	n.r.

^a^Only patients with HCV-1 infection

^b^Bridging fibrosis or fibrosis

^c^Fibrosis stage F3 or F4

^d^No early stopping rules according to study protocol, but within the discretion of the study physician

*EVR* early virologic response, *RVR* rapid virologic response, *n.r.* not reported, *w.a*. weight-adapted (1000 mg for body weight <75 kg; 1200 mg for body weight ≥75 kg)

### Historical comparison – efficacy

**Fig. 2:**
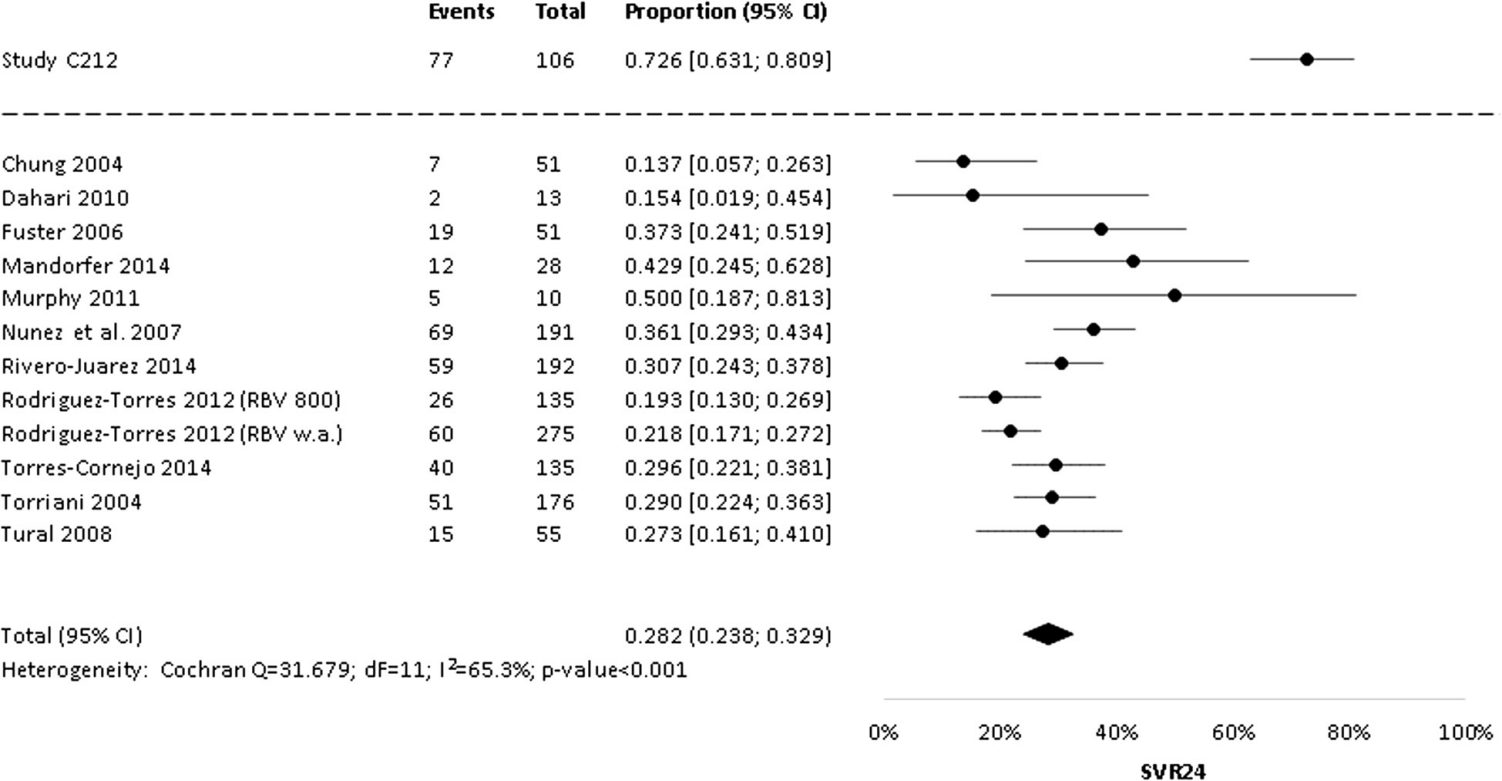
Sustained virologic response in HCV-1/HIV coinfected patients treated with simeprevir + PegIFNα-2a + RBV (study C212) or PegIFNα-2a + RBV alone (random-effects meta-analysis of historical studies). CI = confidence interval; RBV = Ribavirin; SVR24 = sustained virologic response 24 weeks after planned end of treatment; w.a. = weight-adapted

From all included 12 study groups, results on SVR24 were available. The meta-analysis indicated heterogeneity between PegIFNα-2a + RBV study groups (I^2^ = 65.3 %; p < 0.001). However, the results of the fixed-effects model (SVR24 = 27.7 %; 95 % CI 25.3 to 30.2 %) and the random-effects model (SVR24 = 28.2 %; 95 % CI 23.8 % to 32.9 % - see Fig. 2) were similar. After re-transformation of proportions into estimated numerator/denominator pairs (364/1313 for the fixed effects model, and 104/369 for the random effects model; see Additional file 2 for details), the comparison between study C212 (SVR24 = 72.6 %; 95 % CI 63.1 % to 80.9 %) and the meta-analyses results revealed substantial superiority of simeprevir + PegIFNα-2a + RBV compared to PegIFNα-2a + RBV alone (see Table 2), with a relative risk of 2.58 (random effects model) or 2.62 (fixed effects model) and a risk difference of 45 % (both models).

**Table 2:** Main results of historical comparison (simeprevir + PegIFNα-2a + RBV vs. PegIFNα-2a + RBV)

	Number of patients with event/total number of patients (%)		Relative risk (95 % CI)	Risk difference (95 % CI)
	Simeprevir + PegIFNα-2a + RBV	PegIFNα-2a + RBV		
Efficacy				
SVR24 (random effects)	77/106 (72.6 %)	104/369 (28.2 %)	2.58 (2.11; 3.15)	0.45 (0.34; 0.55)
SVR24 (fixed effects)		364/1313 (27.7 %)	2.62 (2.27; 3.03)	0.45 (0.36; 0.54)
Safety				
At least one AE^a^	103/106 (97.2 %)	396/410 (96.6 %)	1.01 (0.97; 1.04)	0.01 (−0.04; 0.05)
At least one SAE^a^	11/106 (10.4 %)	67/407 (16.5 %)	0.63 (0.35; 1.15)	−0.06 (−0.14; 0.01)
AEs leading to discontinuation^b^	5/106 (4.7 %)	21/135 (15.6 %)	0.30 (0.12; 0.78)	−0.11 (−0.19; −0.03)
Anemia AEs (random effects)	35/106 (33.0 %)	58/202 (28.7 %)	1.15 (0.81; 1.63)	0.04 (−0.07; 0.16)
Anemia AEs (fixed effects)		124/418 (29.7 %)	1.11 (0.82; 1.52)	0.03 (−0.07; 0.14)
Psychiatric disorders AEs^c^	63/106 (59.4 %)	3/10 (30.0 %)	1.98 (0.76; 5.17)	0.29 (−0.06; 0.65)

^a^No difference between random and fixed effects models

^b^Results for PegIFNα-2a + RBV from Torres-Cornejo 2014 [18]

^c^Results for PegIFNα-2a + RBV from Murphy 2011 [15]

*CI* confidence interval, *SVR24* sustained virologic response 24 weeks after planned end of treatment, *AE* adverse event, *SAE* serious adverse event, *RBV* ribavirin, *PegIFNα-2a* peginterferon-alpha-2a

The sensitivity analysis that included only studies (*N* = 5) with at least 48 weeks of treatment duration and weight-adapted dosing of RBV revealed similar results (random effects model: RR = 2.59; 95 % CI 2.06 to 3.26 and fixed effects model: RR = 2.70; 95 % CI 2.28 to 3.21). A direct comparison between C212 and the individual historical study groups also showed statistically significant superiority of simeprevir + PegIFNα-2a + RBV for 11 comparisons; for one study [15] the effect estimate was not statistically significant, but this was attributable to the low number of patients included in this study (*N* = 10).

### Historical comparison – safety

The comparison between simeprevir + PegIFNα-2a + RBV and historical data for PegIFNα-2a + RBV did not indicate an increase in risk of the AE categories included (see Table 2). These analyses were limited by the low number of historical studies contributing data.

## Discussion

The historical comparison revealed a substantially higher proportion of patients with SVR with simeprevir + PegIFNα-2a + RBV compared to PegIFNα-2a + RBV alone in patients with HCV-1 and HIV coinfection. The absolute difference in risk was approximately 45 %, which corresponds to a number-needed-to treat of 2.2. No increases in risk were observed for the total number of AEs; serious AEs; AEs leading to discontinuation; anemia AEs; or AEs indicating psychiatric disorders.

For patients with chronic hepatitis due to HCV-1 infection but without HIV, results from RCTs comparing simeprevir with placebo in addition to PegIFNα + RBV are available. These trials demonstrated increased SVR with simeprevir, with absolute differences in risk ranging between approx. 30 % for treatment-naïve patients (QUEST-1 [23] and QUEST-2 [24]) and approx. 45 % for relapsers (PROMISE [25]) or non-responders (ASPIRE [26]) after a previous IFN-based therapy. These differences were primarily due to different proportions of patients with SVR in the placebo groups (i.e. Placebo + PegIFNα + RBV) of these trials. While treatment-naïve patients (QUEST-1 and QUEST-2) had a SVR of approx. 50 %, relapsers (PROMISE) showed a SVR of 36 %, and previous non-responders had a SVR of 23 %. In the meta-analysis for the historical comparison, the overall estimate for PegIFNα-2a + RBV in was 28.2 %, even though included trials only included treatment-naïve patients or did not report on prior treatment status. This is in line with the fact that response to PegIFNα-2a + RBV is worse in patients with HCV/HIV coinfection, compared to HCV infection alone [27].

A recent systematic review of the treatment of hepatitis C [27] identified two RCTs that compared boceprevir or telaprevir with placebo (in addition to PegIFNα + RBV) in patients with chronic hepatitis C who were coinfected with HCV and HIV. The study on boceprevir [6] included 99 treatment-naïve patients with HCV genotype 1 infection and a controlled HIV infection and randomized them 2:1 to boceprevir + PegIFNα-2b + RBV, or to placebo + PegIFNα-2b + RBV. Boceprevir was superior to placebo with respect to SVR24 (absolute difference in risk 33.1 %; 95 % CI 13.7-52.5) but was associated with an increased frequency of AEs such as anemia. Treatment discontinuations due to AEs occurred more frequently with boceprevir than with placebo (20 % vs. 9 %). The proportion of patients with SVR24 in the placebo group (29.4 %) was similar to the proportion of patients with SVR24 in the meta-analysis for the historical comparison (28.2 %). The telaprevir RCT [7] randomized 64 treatment-naïve patients with HCV genotype 1 infection and HIV-1 infection to telaprevir or placebo, both in addition to PegIFNα-2a + RBV. Telaprevir was superior to placebo with respect to SVR24, with an absolute difference in risk of 29 % (95 % CI 3–53). The proportion of patients with SVR24 in the placebo group was 45 % (10 out of 22 patients), however this estimate was limited by the rather low number of patients included. Telaprevir was associated with a significantly increased frequency of pruritus (39 % vs. 9 %), and a non-significant increase in the proportion of patients with at least one serious AE (18 % vs. 9 %).

Compared to simeprevir (1 capsule per day), the pill-burden associated with boceprevir or telaprevir is substantially higher (12 capsules or 6 tablets per day, respectively). In addition, simeprevir seems to have some advantages related to AEs. The frequency of anemia was similar in the simeprevir and the placebo group in four RCTs that included non-HIV patients [23–26]. The historical comparison also did not indicate a higher frequency of anemia in patients treated with simeprevir + PegIFNα-2a + RBV, compared to PegIFNα-2a + RBV alone. No historical comparisons were possible for cutaneous events such as rash and/or pruritus. Data from RCTs in non-HIV patients on cutaneous events associated with simeprevir are heterogeneous. While two studies reported no differences between simeprevir and placebo (PROMISE, QUEST-1), two other trials reported a higher frequency of rash and photosensitivity (QUEST-2) or rash (ASPIRE) in the simeprevir group.

Treatment recommendations for chronic hepatitis C have changed dramatically during the last few years, based on the availability of new direct acting antiviral agents such as telaprevir, boceprevir, simeprevir, sofosbuvir, and daclatasvir. According to current guidelines for the treatment of patients with HCV-1/HIV coinfection published by the American Association for the Study of Liver Diseases (AASLD) [28], simeprevir + PegIFNα-2a + RBV is recommended as an alternative regimen for treatment-naïve or treatment experienced (prior PEG/RBV relapse) HIV/HCV- coinfected patients with genotype 1 who are eligible to receive IFN. The World Health Organization (WHO) Guideline [29] mention PegIFNα-2a + RBV alone or in combination with simeprevir, telaprevir or boceprevir as potential treatment options; and the guideline of the European Association for the Study of the Liver (EASL) [30] recommends simeprevir + PegIFNα-2a + RBV as one of the six treatment options for HCV-1 infected patients. In contrast to earlier findings of lower SVR rates in HIV/HCV- coinfected patients, treatment response to triple therapy or new interferon-free regimens is now comparable to those observed in patients with HCV-monoinfection [30].

For the historical comparison between simeprevir + PegIFNα-2a + RBV and PegIFNα-2a + RBV alone, some methodological limitations have to be considered. One major limitation is that every historical comparison is subject to potential biases that do not exist for randomized head-to-head comparisons. The evidence from such a comparison is thus always considered to be lower than corresponding evidence from RCTs. Study designs and study populations differed between study C212 and the trials included for the historical comparison, as well as among the historical studies. As a consequence, factors associated with the study outcome that differed between study C212 and the historical studies may have confounded the results. No trial was identified that exactly matched the study design of study C212. Main differences were the duration of PegIFNα-2a + RBV treatment and the daily dose of RBV. Because of anticipated drug-drug interactions between commonly used HIV nucleosides and ribavirin, initial trials investigated lower ribavirin doses (600 mg starting dose in the ACTG trial [12], 800 mg in APRICOT [19]). Subsequent European studies [16] showed markedly improved HCV cure rates after using weight adapted ribavirin. This was, however, not demonstrated in the controlled randomized trial which investigated different ribavirin dosages [21], so that the actual influence of ribavirin dose on SVR rates remains unclear. In addition, random effects meta-analyses were used to take into account between-study variation of results. As no meta-regression analysis was performed, no conclusions regarding the impact of potential confounding factors on the outcomes of interest could be made. However, the superiority of simeprevir + PegIFNα-2a + RBV over PegIFNα-2a + RBV alone was not only evident for the comparison with the meta-analysis results, but also for all but one possible comparisons with the individual studies (excluding one study with *N* = 10 participants). In addition, sensitivity analyses were performed for SVR24 in which only trials with a treatment duration at least as long as in study C212 and with the same weight-adapted RBV dose were included. The results were very similar, indicating that these characteristics did not substantially bias the historical comparison for the efficacy measure. Study C212 included patients with previous PegIFNα-2a/RBV treatment, while the historical trials did not or did not report on that aspect. However, for SVR24 this was most likely conservative with respect to the comparison of simeprevir + PegIFNα-2a + RBV with PegIFNα-2a + RBV alone, as SVR24 in study C212 was lower for patients with previous PegIFNα-2a/RBV treatment than for treatment naïve patients. Results regarding AEs from the historical comparison have to be interpreted with special caution, as differences in study design, treatment duration, and/or HIV background therapy may have influenced the proportion of patients with these events. It is difficult to conclude in which direction (i.e. higher or lower risk) the effect measures might were biased by the combination of these study differences. The finding of a lower number of patients who interrupted treatment due to an AE with simeprevir + PegIFNα-2a + RBV compared to PegIFNα-2a + RBV alone was based on one historical study and should be interpreted with caution.

However, the study also has important strengths. The historical comparison was based on a systematic review of available evidence for the treatment efficacy of PegIFNα-2a + RBV in HCV-1/HIV coinfected patients. Meta-analyses methods to combine the available evidence were used instead of using a single study for a historical comparison. The main results for SVR24 did not depend on the type of meta-analysis model used (fixed or random effects) and were stable in sensitivity analyses that tended to underestimate the treatment difference between simeprevir + PegIFNα-2a + RBV and PegIFNα-2a + RBV alone.

## Conclusions

This historical comparison provides first systematic evidence for the superiority of simeprevir + PegIFNα-2a + RBV compared to PegIFNα-2a + RBV alone in patients with HCV-1 and HIV coinfection. The absolute difference of the proportion of patients with SVR24 was approx. 45 %, which is compatible with data from simeprevir RCTs that included non-HIV patients with HCV-1 infection. No increases in the frequency of important AE categories were identified, however these analyses were limited methodologically and by the low number of studies contributing data to these comparisons. Additional data (for instance from observational studies) on the safety of simeprevir triple therapy compared to PegIFNα-2a + RBV alone and compared to other HCV treatment options would be desirable.

## Additional files

Additional file 1: **Electronic search strategy for Medline.** (DOCX 16 kb)

Additional file 2: **Additional data on meta-analyses and historical comparisons.** (DOCX 18 kb)

## Abbreviations

AASLD: American Association for the Study of Liver Diseases
AE: Adverse event
b.i.d.: bis in die = twice a day
C212: TMC435-TiDP16-C212
CI: confidence interval
EASL: European Association for the Study of the Liver
EMBASE: Excerpta Medica Database
HAART: highly active antiretroviral therapy
HCV: hepatitis C virus
HCV-1: hepatitis C virus with genotype 1
HIV-1: human immunodeficiency virus-type 1
IFN: Interferon
PegIFNα: pegylated interferon alfa
PRISMA: Preferred Reporting Items for Systematic Reviews and Meta-Analyses
q.d.: quaque die = once a day
RBV: ribavirin
RCT: randomized controlled trial
SVR: sustained virologic response
SVR12: sustained virologic response 12 weeks after end of treatment
SVR24: sustained virologic response 24 weeks after end of treatment
WHO: World Health Organization
