# Supplementary material for: Simeprevir with pegylated interferon alfa 2a plus ribavirin for treatment of hepatitis C virus genotype 1 in patients with HIV: a meta-analysis and historical comparison
Source: BMC Infect Dis. 2016 Jan 11;16:10. doi: 10.1186/s12879-015-1311-3 (PMC4709957; doi:10.1186/s12879-015-1311-3)
Supplement: Supplementary file 1 — Electronic search strategy for Medline. (DOCX 16 kb) [file 12879_2015_1311_MOESM1_ESM.docx]

**Appendix 1 - Electronic search strategy for Medline**

| **Name of database** | | MEDLINE (ME60) | |
| --- | --- | --- | --- |
| **Date of search** | | July 24, 2014 | |
| **Time period of search** | | 1960 to date of search | |
| **Language** | | English & German | |
| **#** | **Search terms** | | **# of records** |
| 42 | ME60 | | 23,963,834 |
| 43 | CT=HEPATITIS C | | 31,180 |
| 44 | CT=HEPATITIS C, CHRONIC | | 16,154 |
| 45 | CT=HCV ANTIBODIES | | 5,410 |
| 46 | CT=PT-NANBH | | 31,180 |
| 47 | FT=(HEPATITIS C OR HCV OR NON-A, NON-B HEPATITIS OR PT-NANBH) | | 69,162 |
| 48 | 43 TO 47 | | 69,162 |
| 49 | CT=HIV | | 15,976 |
| 50 | CT=HIV 01 | | 63,889 |
| 51 | CT=HIV 02 | | 3,707 |
| 52 | CT=HIV INFECT | | 139,654 |
| 53 | CT=HIV INFECTION | | 139,654 |
| 54 | CT=HUMAN IMMUNODEFICIENCY VIRUS | | 15,976 |
| 55 | CT=HUMAN IMMUNODEFICIENCY VIRUS 1 | | 63,889 |
| 56 | CT=HUMAN IMMUNODEFICIENCY VIRUS 2 | | 3,707 |
| 57 | CT=HUMAN IMMUNODEFICIENCY VIRUS TYPE 1 | | 63,889 |
| 58 | CT=HUMAN IMMUNODEFICIENCY VIRUS TYPE 2 | | 3,707 |
| 59 | CT=HUMAN IMMUNODEFICIENCY VIRUSES | | 15,976 |
| 60 | CT=AIDS | | 72,228 |
| 61 | CT=ACQUIRED IMMUNE DEFICIENCY SYNDROME | | 72,228 |
| 62 | FT=(HIV OR HUMAN IMMUNODEFICIENCY VIRUS## OR AIDS OR ACQUIRED IMMUNE DEFICIENCY SYNDROME) | | 348,966 |
| 63 | 49 TO 62 | | 358,666 |
| 64 | 48 AND 63 | | 12,220 |
| 65 | FT=(HIV-HCV, # # # COINFECT? OR HIV-HCV, # # # COINFECT?) | | 1,354 |
| 66 | 64 OR 65 | | 12,220 |
| 67 | TE=PEGINTERFERON ALFA-2A | | 2,997 |
| 68 | TE=PEGINTERFERON ALFA-2B | | 2,024 |
| 69 | FT=(PEGINTERFERON? OR PEG-INTERFERON?) | | 5,093 |
| 70 | FT=(PEG-IFN? OR PEGIFN?) | | 2,042 |
| 71 | FT=(PEGYLATED INTERFERON? OR PEGYLATED IFN?) | | 4,603 |
| 72 | FT=(PEGASYS OR PEGINTRON OR PEG-INTRON) | | 154 |
| 73 | 67 TO 72 | | 7,560 |
| 74 | CT=RIBAVIRIN | | 8,361 |
| 75 | CT=RIBAVIRIN MERCK BRAND | | 8,361 |
| 76 | TE=RIBAVIRIN | | 8,361 |
| 77 | FT=RIBAVIRIN | | 11,961 |
| 78 | FT=(TRIBAVIRIN OR RIBOVIRIN OR REBETOL OR VIRAZOLE OR VILONA OR RIBASPHERE OR VIRAMIDE OR VIRAZIDE OR RIBAMIDE OR RIBAMIDYL OR RIBAMIDIL OR RIBAVIRINE OR ICN-1229 OR ICN1229) | | 273 |
| 79 | 74 TO 78 | | 12,029 |
| 80 | 73 AND 79 | | 5,820 |
| 81 | FT=(PEG-IFN-RBV OR PEGIFN-RBV OR PEGINTERFERON-RIBAVIRIN OR PEG-INTERFERON-RIBAVIRIN OR PEGYLATED INTERFERON-RIBAVIRIN OR PEGYLATED IFN-RIBAVIRIN) | | 650 |
| 82 | 80 OR 81 | | 5,831 |
| 83 | 66 AND 82 | | 862 |
| 84 | 83 AND PPS=HUMAN | | 859 |
| 85 | 84 AND LA=(ENGL; GERM) | | 795 |
| 86 | DT=CONTROLLED CLINICAL TRIAL | | 87,522 |
| 87 | DT=RANDOMIZED CONTROLLED TRIAL | | 36,8229 |
| 88 | CT=CLINICAL TRIALS, CONTROLLED AS TOPIC | | 4,905 |
| 89 | CT=CLINICAL TRIALS, RANDOMIZED | | 90,835 |
| 90 | CT=CONTROLLED CLINICAL TRIALS AS TOPIC | | 4,905 |
| 91 | CT=CONTROLLED CLINICAL TRIALS, RANDOMIZED | | 90,835 |
| 92 | CT=RANDOMIZED CONTROLLED TRIALS AS TOPIC | | 90,835 |
| 93 | CT=RANDOMIZATION | | 80,009 |
| 94 | CT=DOUBLE BLIND STUDY | | 123,914 |
| 95 | DT=CLINICAL TRIAL, PHASE II | | 22,245 |
| 96 | DT=CLINICAL TRIAL, PHASE III | | 8,697 |
| 97 | DT=CLINICAL TRIAL, PHASE IV | | 900 |
| 98 | CT=CLINICAL TRIALS, PHASE II AS TOPIC | | 5,736 |
| 99 | CT=CLINICAL TRIALS, PHASE III AS TOPIC | | 6,185 |
| 100 | CT=CLINICAL TRIALS, PHASE IV AS TOPIC | | 217 |
| 101 | CT=CLINICAL TRIALS, PHASE 2 AS TOPIC | | 0 |
| 102 | CT=CLINICAL TRIALS, PHASE 3 AS TOPIC | | 6,185 |
| 103 | CT=CLINICAL TRIALS, PHASE 4 AS TOPIC | | 217 |
| 104 | CT=CLINICAL TRIAL# AS TOPIC AND FT=(PHASE II OR PHASE III OR PHASE IV OR PHASE 2 OR PHASE 3 OR PHASE 4) | | 6,068 |
| 105 | FT=(TRIAL# AND (PHASE II OR PHASE III OR PHASE IV OR PHASE 2 OR PHASE 3 OR PHASE 4))/SAME SENT | | 59,733 |
| 106 | FT=(STUD### AND (PHASE II OR PHASE III OR PHASE IV OR PHASE 2 OR PHASE 3 OR PHASE 4))/SAME SENT | | 40,225 |
| 107 | FT=(RANDOM? OR CONTROLLED? OR PLACEBO? OR DOUBLE-BLIND? OR OPEN-LABEL? OR OPEN STUD?) | | 1,326,428 |
| 108 | (95 TO 106) AND 107 | | 30,558 |
| 109 | (86 TO 94) OR 108 | | 619,807 |
| 110 | RANDOMIZED CONTROLLED TRIAL/DT | | 368,212 |
| 111 | RANDOMIZED/(TI; AB; CT) | | 376,921 |
| 112 | PLACEBO/(TI; AB; CT) | | 159,365 |
| 113 | 110 TO 112 | | 614,524 |
| 114 | 85 AND 109 | | 99 |
| 115 | 85 AND 113 | | 119 |
| 116 | 114 OR 115 | | 125 |
| 117 | (TRIAL# OR STUD###)/(TI; AB; CT; DT) | | 8,993,465 |
| 118 | 85 AND 117 | | 570 |
| 119 | 118 NOT 116 | | 445 |
| 120 | 116 OR 119 | | 570 |
