# Supplementary material for: Simeprevir with pegylated interferon alfa 2a plus ribavirin for treatment of hepatitis C virus genotype 1 in patients with HIV: a meta-analysis and historical comparison
Source: BMC Infect Dis. 2016 Jan 11;16:10. doi: 10.1186/s12879-015-1311-3 (PMC4709957; doi:10.1186/s12879-015-1311-3)
Supplement: Supplementary file 2 — Additional data on meta-analyses and historical comparisons. (DOCX 18 kb) [file 12879_2015_1311_MOESM2_ESM.docx]

**Appendix 2 – Additional data on meta-analyses and historical comparisons**

**Table A2-1. Meta analyses „Sustained virologic response 24 weeks after planned end of treatment“**

| **Study/Analysis** | **Statistic** | **Result** |
| --- | --- | --- |
| Chung 2004 | Proportion | 7/51 (13.7 %) |
| Dahari 2010 | Proportion | 2/13 (15.4 %) |
| Fuster 2006 | Proportion | 19/51 (37.3 %) |
| Mandorfer 2014 | Proportion | 12/28 (42.9 %) |
| Murphy 2011 | Proportion | 5/10 (50.0 %) |
| Nunez et al. 2007 | Proportion | 69/191 (36.1 %) |
| Rivero-Juarez 2014 | Proportion | 59/192 (30.7 %) |
| Rodriguez-Torres 2012 (RBV 800) | Proportion | 26/135 (19.3 %) |
| Rodriguez-Torres 2012 (RBV WB) | Proportion | 60/275 (21.8 %) |
| Torres-Cornejo 2014 | Proportion | 40/135 (29.6 %) |
| Torriani 2004 | Proportion | 51/176 (29.0 %) |
| Tural 2008 | Proportion | 15/55 (27.3 %) |
|  |  |  |
| Fixed effects Meta-Analysis | Proportion (95 % CI) | 0.277 (0.253; 0.302) |
|  | Cochran Q. dF | 31.679; dF=11 |
|  | p-value (Heterog.) | <0.001 |
|  | I (Heterog.) | 65.3% |
|  | Iterative: combined number of events/total | 364/1313 |
|  | Iterative: actual proportion (95 % CI) | 0.277 (0.253; 0.302) |
|  |  |  |
| Random effects Meta-Analysis | Proportion (95 % CI) | 0.282 (0.238; 0.329) |
|  | Iterative: combined number of events/total | 104/369 |
|  | Iterative: actual proportion (95 % CI) | 0.282 (0.236; 0.331) |

**Table A2-2. Meta analyses** **„Proportion of patients with at least one adverse event“**

| **Study/Analysis** | **Statistic** | **Result** |
| --- | --- | --- |
| Rodriguez-Torres 2012 (RBV 800) | Proportion | 132/135 (97.8 %) |
| Rodriguez-Torres 2012 (RBV WB) | Proportion | 264/275 (96.0 %) |
|  |  |  |
| Fixed effects Meta-Analysis | Proportion (95 % CI) | 0.965 (0.945; 0.981) |
|  | Cochran Q. dF | 0.729; dF=1 |
|  | p-value (Heterog.) | 0.393 |
|  | I (Heterog.) | 0.0% |
|  | Iterative: combined number of events/total | 396/410 |
|  | Iterative: actual proportion (95 % CI) | 0.966 (0.943; 0.981) |
|  |  |  |
| Random effects Meta-Analysis | Proportion (95 % CI) | 0.965 (0.945; 0.981) |
|  | Iterative: combined number of events/total | 396/410 |
|  | Iterative: actual proportion (95 % CI) | 0.966 (0.943; 0.981) |

**Table A2-3. Meta analyses „Proportion of patients with at least one serious adverse event“**

| **Study/Analysis** | **Statistic** | **Result** |
| --- | --- | --- |
| Murphy 2011 | Proportion | 2/10 (20.0 %) |
| Rodriguez-Torres 2012 (RBV 800) | Proportion | 33/135 (24.4 %) |
| Rodriguez-Torres 2012 (RBV WB) | Proportion | 89/275 (32.4 %) |
|  |  |  |
| Fixed effects Meta-Analysis | Proportion (95 % CI) | 0.296 (0.253; 0.340) |
|  | Cochran Q. dF | 2.996. dF=2 |
|  | p-value (Heterog.) | 0.224 |
|  | I (Heterog.) | 33.3% |
|  | Iterative: combined number of events/total | 124/418 |
|  | Iterative: actual proportion (95 % CI) | 0.297 (0.253; 0.343) |
|  |  |  |
| Random effects Meta-Analysis | Proportion (95 % CI) | 0.288 (0.228; 0.351) |
|  | Iterative: combined number of events/total | 58/202 |
|  | Iterative: actual proportion (95 % CI) | 0.287 (0.226; 0.355) |

**Table A2-4. Meta analyses „Proportion of patients with at least one adverse event of anemia“**

| **Study/Analysis** | **Statistic** | **Result** |
| --- | --- | --- |
| Rodriguez-Torres 2012 (RBV 800) | Proportion | 21/135 (15.6 %) |
| Rodriguez-Torres 2012 (RBV WB) | Proportion | 46/275 (16.7 %) |
|  |  |  |
| Fixed effects Meta-Analysis | Proportion (95 % CI) | 0.164 (0.130; 0.202) |
|  | Cochran Q. dF | 0.072. dF=1 |
|  | p-value (Heterog.) | 0.788 |
|  | I (Heterog.) | 0.0% |
|  | Iterative: combined number of events/total | 67/407 |
|  | Iterative: actual proportion (95 % CI) | 0.165 (0.130; 0.204) |
|  |  |  |
| Random effects Meta-Analysis | Proportion (95 % CI) | 0.164 (0.130; 0.202) |
|  | Iterative: combined number of events/total | 67/407 |
|  | Iterative: actual proportion (95 % CI) | 0.165 (0.130; 0.204) |

**Table A2-5. Sensitivity analysis - Meta analyses „Sustained virologic response 24 weeks after planned end of treatment“, only including studies with at least 48 weeks planned duration of treatment and weight-adapted dosing of ribavirin**

| **Study/Analysis** | **Statistic** | **Result** |
| --- | --- | --- |
| Murphy 2011 | Proportion | 5/10 (50.0 %) |
| Rivero-Juarez 2014 | Proportion | 59/192 (30.7 %) |
| Rodriguez-Torres 2012 (RBV WB) | Proportion | 60/275 (21.8 %) |
| Torres-Cornejo 2014 | Proportion | 40/135 (29.6 %) |
| Tural 2008 | Proportion | 15/55 (27.3 %) |
|  |  |  |
| Fixed effects Meta-Analysis | Proportion (95 % CI) | 0.268 (0.235; 0.303) |
|  | Cochran Q. dF | 8.151; dF=4 |
|  | p-value (Heterog.) | 0.086 |
|  | I (Heterog.) | 50.9% |
|  | Iterative: combined number of events/total | 179/666 |
|  | Iterative: actual proportion (95 % CI) | 0.269 (0.235; 0.304) |
|  |  |  |
| Random effects Meta-Analysis | Proportion (95 % CI) | 0.279 (0.226; 0.335) |
|  | Iterative: combined number of events/total | 72/257 |
|  | Iterative: actual proportion (95 % CI) | 0.280 (0.226; 0.339) |

**Table A2-6. Sensitivity analysis for historical comparison for „Sustained virologic response 24 weeks after planned end of treatment“ (simeprevir+PegIFNα-2a+RBV vs. PegIFNα-2a+RBV)**

| **Type of meta-analysis** | **Study C212** | **Data from meta-analysis** | **RR (95 % CI)** | **RD (95 % CI)** |
| --- | --- | --- | --- | --- |
| Fixed effects | 77/106 (72.6 %) | 179/666 (26.9 %) | 2.703 (2.277; 3.208) | 0.458 (0.361; 0.554) |
| Random effects |  | 72/257 (28.0 %) | 2.593 (2.064; 3.257) | 0.446 (0.339; 0.554) |

**Table A2-7. Historical comparison of study C212 with all individual studies / study groups**

| **Endpoint** | **Data source for historical comparison** | **Study C212** | **Historical study** | **RR (95%CI)** | **RD (95%CI)** |
| --- | --- | --- | --- | --- | --- |
| SVR24 | Chung 2004 | 77/106 (72.6 %) | 7/51 (13.7 %) | 5.292 (2.634; 10.636) | 0.589 (0.448; 0.731) |
|  | Dahari 2010 | 77/106 (72.6 %) | 2/13 (15.4 %) | 4.722 (1.313; 16.985) | 0.573 (0.316; 0.829) |
|  | Fuster 2006 | 77/106 (72.6 %) | 19/51 (37.3 %) | 1.950 (1.340; 2.837) | 0.354 (0.182; 0.526) |
|  | Mandorfer 2014 | 77/106 (72.6 %) | 12/28 (42.9 %) | 1.695 (1.088; 2.641) | 0.298 (0.073; 0.522) |
|  | Murphy 2011 | 77/106 (72.6 %) | 5/10 (50.0 %) | 1.453 (0.773; 2.730) | 0.226 (-0.150; 0.602) |
|  | Nunez et al. 2007 | 77/106 (72.6 %) | 69/191 (36.1 %) | 2.011 (1.611; 2.510) | 0.365 (0.249; 0.481) |
|  | Rivero-Juarez 2014 | 77/106 (72.6 %) | 59/192 (30.7 %) | 2.364 (1.855; 3.012) | 0.419 (0.305; 0.534) |
|  | Rodriguez-Torres 2012 (RBV 800) | 77/106 (72.6 %) | 26/135 (19.3 %) | 3.772 (2.619; 5.431) | 0.534 (0.418; 0.650) |
|  | Rodriguez-Torres 2012 (RBV WB) | 77/106 (72.6 %) | 60/275 (21.8 %) | 3.329 (2.587; 4.285) | 0.508 (0.404; 0.613) |
|  | Torres-Cornejo 2014 | 77/106 (72.6 %) | 40/135 (29.6 %) | 2.452 (1.844; 3.260) | 0.430 (0.307; 0.553) |
|  | Torriani 2004 | 77/106 (72.6 %) | 51/176 (29.0 %) | 2.507 (1.935; 3.248) | 0.437 (0.321; 0.552) |
|  | Tural 2008 | 77/106 (72.6 %) | 15/55 (27.3 %) | 2.664 (1.703; 4.165) | 0.454 (0.295; 0.613) |
| Patients with at least one AE | Rodriguez-Torres 2012 (RBV 800) | 103/106 (97.2 %) | 132/135 (97.8 %) | 0.994 (0.954; 1.036) | -0.006 (-0.055; 0.043) |
|  | Rodriguez-Torres 2012 (RBV WB) | 103/106 (97.2 %) | 264/275 (96.0 %) | 1.012 (0.972; 1.054) | 0.012 (-0.034; 0.057) |
| Patients with at least one SAE | Rodriguez-Torres 2012 (RBV 800) | 11/106 (10.4 %) | 21/135 (15.6 %) | 0.667 (0.337; 1.322) | -0.052 (-0.145; 0.041) |
|  | Rodriguez-Torres 2012 (RBV WB) | 11/106 (10.4 %) | 46/275 (16.7 %) | 0.620 (0.334; 1.152) | -0.063 (-0.143; 0.016) |
| Patients with AE leading to discont. | Torres-Cornejo 2014 | 5/106 (4.7 %) | 21/135 (15.6 %) | 0.303 (0.118; 0.777) | -0.108 (-0.190; -0.027) |
| Patients with anemia | Murphy 2011 | 35/106 (33.0 %) | 2/10 (20.0 %) | 1.651 (0.464; 5.872) | 0.130 (-0.188; 0.448) |
|  | Rodriguez-Torres 2012 (RBV 800) | 35/106 (33.0 %) | 33/135 (24.4 %) | 1.351 (0.904; 2.019) | 0.086 (-0.038; 0.209) |
|  | Rodriguez-Torres 2012 (RBV WB) | 35/106 (33.0 %) | 89/275 (32.4 %) | 1.020 (0.740; 1.406) | 0.007 (-0.105; 0.118) |
| Patients with psychiatric AEs | Murphy 2011 | 63/106 (59.4 %) | 3/10 (30.0 %) | 1.981 (0.759; 5.173) | 0.294 (-0.059; 0.648) |
